# An Account of the Good Effects of Electricity in a Case of Paralytic Affection; Serving to Prove That, in Such Cases, the Electric Sparks Should Be Taken from the Muscles Which Are Antagonists to Those That Are Contracted

**Published:** 1792

**Authors:** William Gilby

**Affiliations:** Physician to the General Hospital at Birmingham.


IX. *An Account of the good Effects of Electricity in a Case of paralytic Affection; serving to prove that, in such Cases, the electric Sparks should be taken from the Muscles which are Antagonists to those that are contracted. Communicated in a Letter to Dr.* Simmons *by* William Gilby, *M. D. Physician to the General Hospital at Birmingham.*

I SEND you the following case as an additional proof, to that which I communicated to you last year *, of the good effects of electricity in muscular contractions.

It has hitherto been the practice of medical men to use electricity to the contracted muscles, which is a practice I wish to see laid aside; its effects being to make the muscular fibres contract more strongly: whereas if the electrical sparks be taken from the muscles which are antagonists to those that are contracted, they prove a very powerful and efficacious remedy.

This is a fact which I am desirous of establishing, and this can only be done by a publication of successful cases.

* See the London Medical Journal, Vol. XI. page 385.

Mr.

Mr. W——, aged about thirty years, was feized, on the 7th of January laft, with a flight paralytic affection. His mouth was drawn to the left fide; his fpeech affected, and it was with difficulty he could take in liquids.

I faw him on the 10th, and ordered a blifter to be applied to the fpine of the neck; and alfo fome pills compofed of camphor and affafœtida, and a mixture confifting principally of *aq. menth. pip.* and *tinct. valer. vol.* Proper dofes of thefe medicines were taken every four or five hours for three days, and the blifter difcharged plentifully, but without being productive of much amendment. I therefore refolved upon drawing electrical fparks from the mufcles inferted into the right angle of the mouth. After this had been done twice a day, for three or four days, the patient had fome power in counteracting the action of the mufcles which were contracted. He faid he could fpeak and take in liquids with much greater eafe.

On the 19th there was a very vifible alteration for the better. He could bring the relaxed mufcles into action at pleafure, but could not make them contract fo far as for the mouth to regain its proper place.

I ſaw him on the 21ſt, 23d, and 25th, and was witneſs to a progreſſive amendment.

On the 2d of February he had nearly a complete command over the relaxed muſcles, ſo that he could draw the mouth as much to the right ſide as to the left.

In this, and alſo in the caſe of Mrs. G——, inſerted in the London Medical Journal, and which I have already referred to, it ſeems a difficult matter to aſcertain whether the diſeaſe depended upon an increaſed power of action in the contracted muſcles, or a want of the ordinary power of action in their antagoniſts.

P. S. Two caſes have lately occurred to me; the one of Samuel Locker, and the other of Emanuel Parſons, in both of which there had been a perfect paralyſis of the extenſor muſcles of the fingers for ſome time. The latter of theſe is quite cured, and the other nearly ſo, by drawing electrical ſparks from theſe muſcles night and morning. The ſkin, in both caſes, was generally much inflamed by the operation, and often bliſtered. Emanuel Parſons had been admitted into two of the hoſpitals in London, but without receiving any advantage whatever.

These men are both of them glafs cutters, and have been accuftomed to work many hours in the day with their hands in cold water.

*Birmingham*,

November 30th, 1791.

